# Evaluating the Efficacy of Large Language Models for Dizzy History Taking and Peripheral Vestibular Disorder Diagnosis

**DOI:** 10.1177/19160216251377349

**Published:** 2025-10-01

**Authors:** Bonnie Lu, Ana-Maria Misariu, Jennifer Inseon Ham, Jason Archibald, Benjamin van der Woerd

**Affiliations:** 1 Michael G. DeGroote School of Medicine, McMaster University, Hamilton, ON, Canada; 2 Division of Otolaryngology—Head & Neck Surgery, Department of Surgery, McMaster University, Hamilton, ON, Canada

**Keywords:** large language models, artificial intelligence, natural language processing, vertigo, otology/neurotology, peripheral vestibular disorders

## Abstract

**Importance:**

Vertigo accounts for one of the most frequent presenting symptoms in primary care. However, complexities in differential diagnoses and reliance on clinical history contribute to frequent specialist referrals and diagnostic delays. Large language models (LLMs), like LLaMA-3.1-8B, offer new potential for assisting in clinical decision-making.

**Objective:**

To assess the utility of a small-scale, open-source LLM in diagnosing peripheral vestibular disorders (PVDs), and evaluate the impact of synthetic data augmentation on diagnostic accuracy.

**Design:**

Retrospective chart review.

**Setting/Participants:**

A retrospective analysis included adult patients presenting with dizziness to a neuro-otologist at St. Joseph’s Healthcare Hamilton between 2018 and 2023. The dataset comprised 100 clinical cases, supplemented with 40 synthetic cases generated using GPT-4. The LLaMA-3.1-8B model was evaluated on the clinical, synthetic, and combined datasets. Diagnostic reasoning approaches, including chain-of-thought reasoning and multi-shot prompting, were employed to optimize model performance.

**Main Outcome Measures:**

Metrics for evaluation included top 1 and top 3 diagnostic accuracy, Cohen’s kappa for inter-rater agreement, and accuracy in predicting symptom laterality.

**Results:**

The LLaMA-3.1-8B model achieved a top 1 diagnostic accuracy of 60.7% and a top 3 accuracy of 71.4% in the combined dataset. The most frequent diagnosis was Meniere’s disease (55.7%), followed by vestibular migraines (9.3%) and labyrinthitis (9.3%). Diagnostic accuracy was highest for benign paroxysmal positional vertigo (90%), followed by Meniere’s disease (80.8%). Less common conditions, such as superior canal dehiscence syndrome and vestibular paroxysmia, exhibited lower diagnostic accuracies. Cohen’s kappa indicated substantial agreement for symptom side prediction (κ = 0.96) and moderate agreement for diagnosis (κ = 0.41) in the combined dataset.

**Conclusions and Relevance:**

The LLaMA-3.1-8B model demonstrated promising accuracy in diagnosing PVDs. The model’s performance highlights its potential to serve as a high-yield screening tool for primary care physicians and general otolaryngologists.

## Graphical abstract

**Graphical Abstract:**
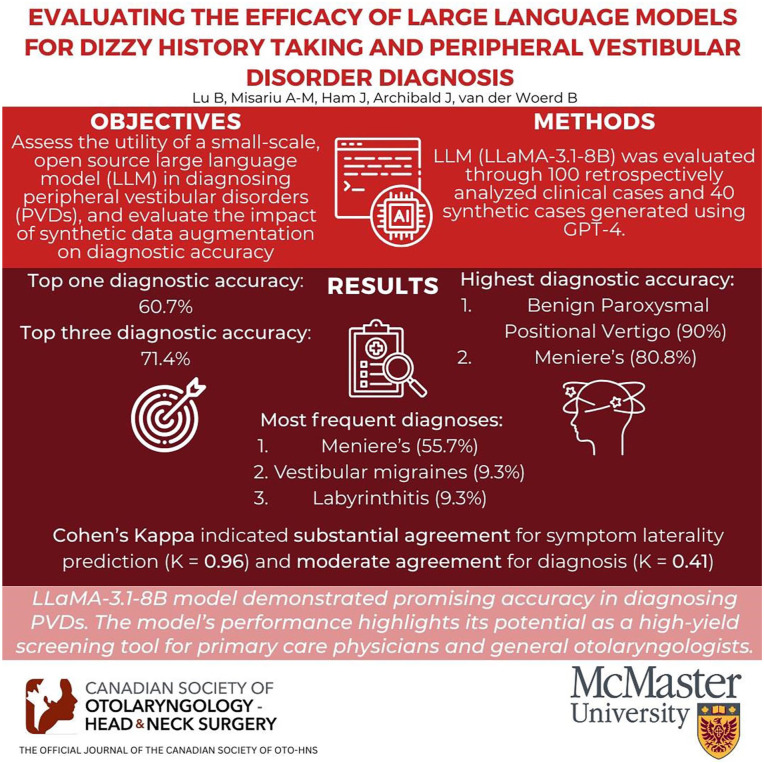

## Introduction

Vertigo, characterized by illusory self-motion, accounts for approximately 1.8% to 4% of presenting symptoms in primary care clinics and emergency departments.^1-4^ Despite its prevalence, prompt diagnosis and management continue to pose a challenge. This difficulty is partly due to a broad list of differential diagnoses, a diagnostic process that relies heavily on clinical history, and a high degree of variability in patients’ symptoms.^5^ These factors contribute to diagnostic delays and frequent referrals to otolaryngologists. Given the long wait times and limited availability of specialists, there is an emerging need for more accessible diagnostic tools to facilitate timely and accurate assessment.^6^

Recent advancements in large language models (LLMs) have led to many medical applications, including those within the field of otolaryngology—head and neck surgery. While LLMs have demonstrated their capabilities in passing medical licensing exams such as the Canadian Royal College of Physicians and Surgeons OHNS certification exam,^7^ it is unclear whether they can be effectively integrated by healthcare professionals in real-world clinical practice.^8-13^ Recent research has highlighted their potential in clinical tasks, such as summarizing medical records and assisting in diagnostic decision-making.^14,15^ However, many of these studies have focused on closed-source models (eg, GPT-4, Gemini), which pose privacy and data security concerns when used with patient data. To date, there are a few, if any, studies applying an open-source LLM to problems of differential diagnosis, and none in the context of vertigo.^16,17^ This study seeks to develop a pipeline for diagnosing peripheral vestibular disorders (PVDs) that can be applied in the clinical setting. By using a small-scale open-source LLM (LLaMA-3.1-8B), we aim to demonstrate the capabilities of a locally run diagnostic tool. Furthermore, we investigate the application of synthetic data augmentation to mitigate biases in dataset composition, a critical consideration when training and evaluating LLMs with limited datasets. We believe that this approach will not only mitigate privacy concerns but also offer a practical solution for enhancing diagnostic accuracy and efficiency in the community-based management of vertigo.

## Methods

### Data Sources and Preparation

This study employs a retrospective analysis approach to evaluate the effectiveness of a small-scale open-source LLM (LLaMA-3.1-8B) in diagnosing PVDs. Adult patients who presented with a primary complaint of dizziness and were seen by a neuro-otologist at St. Joseph’s Healthcare Hamilton between January 2018 and December 2023 were included in the dataset. Patients were excluded if they had incomplete histories or indeterminate diagnoses at the conclusion of their first consultation. The history of present illness, age, sex, and diagnosis were extracted for the first 100 patients who met these criteria. Given inherent biases in dataset composition, arising from overrepresentation of certain diagnoses, synthetic data augmentation was explored to enhance diversity.

Synthetic data augmentation involves the use of LLMs in generating synthetic cases, broadening training data diversity without necessitating further data collection efforts. Previous research has shown promise in using synthetic data to improve model performance and generalizability.^18-20^ We prompted GPT-4.0 with PDFs of the Bárány Society Criteria as reference criteria and provided three extracted clinical prompts for multi-shot prompting to guide formatting. GPT-4.0 then generated a medical history and a ranked list of top 4 differential diagnoses for each case. Two otolaryngologists independently evaluated the synthetic cases for accuracy and clinical relevance. The evaluations focused on the coherence and plausibility of the medical histories and the appropriateness of the differential diagnoses. The validated synthetic data was combined with the original clinical dataset to produce a new dataset of 140 cases. An additional set of 50 synthetic cases was generated using the same methodology to specifically evaluate the model’s ability to distinguish between central and peripheral causes of vertigo. These cases were kept separate from the primary dataset and used exclusively for classification testing.

### Model Specifications

LLaMA-3.1-8B is an open-source LLM developed as part of the LLaMA-3.1 family. Released in July 2024, the LLaMA-3.1 model is one of the largest open-source LLMs to date, rivaling closed-source models such as GPT-4, GPT-4o, and Claude 3.5 Sonnet in its performance and capabilities.^21^ The choice of LLaMA-3.1-8B for this study was guided by several considerations, including computational efficiency and resource constraints. The 8B parameter model requires significantly less computational power and memory compared to its larger counterparts (70B and 405B), making it feasible to run on standard hardware setups without the need for specialized high-performance computing resources. Provided our goal of integrating the tool into clinical workflows, its relatively small size and computational requirements make it an ideal candidate for deployment in resource-constrained clinical environments.

### System Architecture and Techniques

This study explored the development of a diagnostic model using LLaMA-3.1-8B. The model was initially fine-tuned with criteria from Bárány’s International Classification of Vestibular Diseases (ICVD)^22-39^ and cases from the clinical dataset. However, overfitting was observed, due to the homogeneity of the dataset for Meniere’s disease. To mitigate this, we transitioned to using the instruct-tuned LLaMA-3.1-8B model without further fine-tuning on the clinical data. Diagnostic reasoning techniques, such as chain-of-thought prompting, retrieval augmented generation, and multi-shot prompting, were employed to enhance model performance.

**Figure 1.**
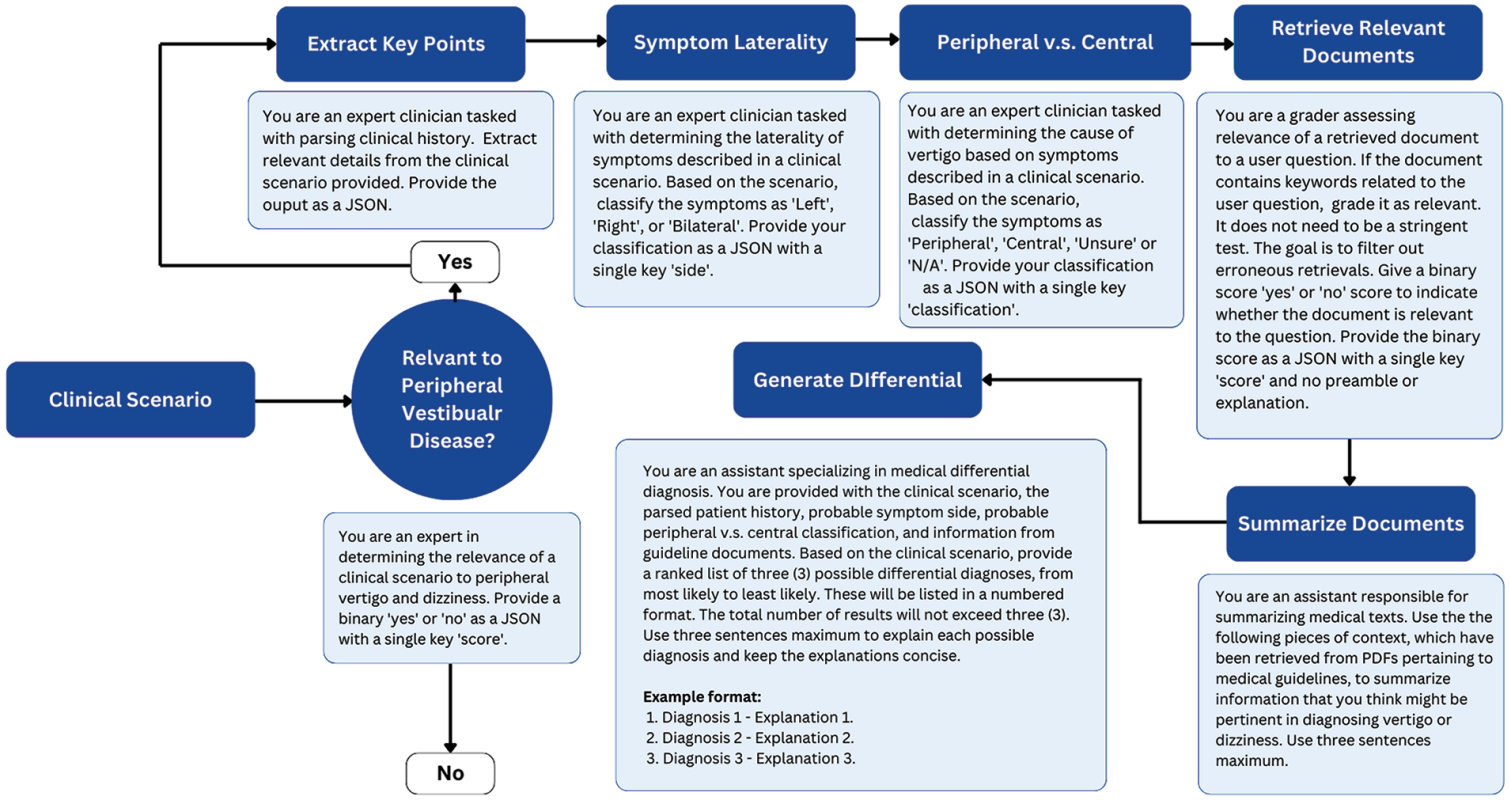
Workflow for the differential diagnosis of peripheral vestibular diseases.

Several studies have demonstrated significant improvements in the performance of LLMs when applying techniques like chain-of-thought prompting and multi-shot prompting.^40-42^ Chain-of-thought prompting, which utilizes intermediate reasoning steps, has been shown to enhance LLM performance in tasks requiring arithmetic, commonsense, and symbolic reasoning. In this study, the differential diagnostic process was dissected into smaller, more manageable tasks. These tasks included extracting relevant information from a general history, determining the relevance of the case to the model’s task, assessing the applicability of the ICVD consensus documents to the clinical scenario, evaluating for symptom side, evaluating for central compared to peripheral etiology, and generating a reasoned list of differential diagnoses. Multi-shot prompting was also utilized, with the model being provided examples of input-output pairs. This allowed the model to learn from context, improving its ability to generalize and reason about unseen cases. Figure 1 illustrates the specific prompts used, including the input, intermediate steps, and output formats. These techniques help decompose complex tasks into manageable steps, enhancing the model’s diagnostic reasoning.

### Evaluation Metrics

Descriptive statistics and inter-rater agreement analyses were done to evaluate the performance of the LLM. We compared the model’s performance, trained on the clinical dataset alone, with its performance on a combination of the clinical dataset and the synthetic dataset. Specifically, we assessed for top 1 and top 3 accuracy, which quantifies the proportion of cases where the model’s top 1 and 3 predictions match the neuro-otologist’s diagnosis, respectively. Cohen’s kappa was calculated to quantify the level of agreement between the neuro-otologist and the model, examining agreement for symptom side and diagnosis.

### Safety Measures and Guardrails

There is increasing concern regarding the protection of patient privacy in the applications of artificial intelligence in health care.^15,43^ This study employed a rigorous approach to address these concerns, incorporating data anonymization, model-selection, and guardrail-constrained modeling. All the identifiable patient information was removed during the extraction of clinical histories. Processing was conducted locally on an open-source model (LLaMA-3.1-8B), providing a more secure alternative to closed-source models (eg, GPT-4.0), which require cloud-based training and pose privacy and security risks. These constraints ensured that the model remained within acceptable and safe boundaries, preventing the generation of harmful, biased, or inappropriate responses.

## Results

### Patient Demographic and Characteristics of the Data

Table 1 summarizes the patient demographic and characteristics of the clinical, synthetic, and combined datasets. The combined dataset (n = 140) had a mean age of 53.2 years (SD = 15.0), which was slightly lower than the clinical dataset’s mean age of 56.6 years (SD = 15.2) and higher than the synthetic dataset’s mean of 44.8 years (SD = 10.5). Sex distribution was consistent across datasets, with males representing 40% in the clinical dataset, 50% in the synthetic dataset, and 42.9% in the combined dataset.

**Table 1.** Patient Demographics and Characteristics of the Clinical, Synthetic, and Combined Datasets.

Patient characteristics	Clinical dataset (N = 100)	Synthetic dataset (N = 40)	Combined dataset (N = 140)
Mean age (SD)	56.6 (15.2)	44.8 (10.5)	53.2 (15.0)
Sex (%)			
Male	40 (40)	20 (50)	60 (42.9)
Female	60 (60)	20 (50)	80 (57.1)
Symptom side (%)			
Right	52 (52)	12 (30)	64 (45.7)
Left	44 (44)	12 (30)	59 (42.1)
Bilateral	4 (4)	16 (40)	17 (12.1)
Diagnosis (%)			
BPPV	1 (1)	9 (22.5)	10 (7.1)
Labyrinthitis	13 (13)	N/A	13 (9.3)
MdDS	N/A	7 (17.5)	7 (5)
Meniere’s disease	78 (78)	N/A	78 (55.7)
SSCD	1 (1)	3 (7.5)	4 (2.9)
Vestibular migraines	1 (1)	12 (30)	13 (9.3)
Vestibular paroxysmia	N/A	9 (22.5)	9 (6.4)
Other	6 (6)	0 (0)	6 (4.3)

Abbreviation: BPPV, benign paroxysmal positional vertigo; MdDS, Mal de Debarquement Syndrome; N/A, not applicable; SSCD, superior semicircular canal dehiscence.

Differences were observed in symptom side distribution. Bilateral symptoms were more common in the synthetic dataset (40%) compared to the clinical dataset (4%). Right- and left-side symptoms were evenly distributed in the synthetic dataset (30% each), whereas the clinical dataset showed a slight right-side preference (52% right, 44% left). In the combined dataset, right- and left-side symptoms were nearly equal, at 45.7% and 42.1%, respectively, with bilateral symptoms accounting for 12.1%.

Diagnoses varied between the datasets. Meniere’s disease was most prevalent in the clinical dataset (78%) but absent in the synthetic data. Conversely, conditions like benign paroxysmal positional vertigo (BPPV) (22.5%) and vestibular migraines (30%) were more frequent in the synthetic dataset, whereas Meniere’s was absent. In the combined dataset, Meniere’s disease remained the most frequent diagnosis (55.7%), followed by Vestibular Migraines (9.3%). Labyrinthitis, most often presenting with vertigo accompanied by ear pain, hearing loss, and tinnitus,^22^ accounted for 9.3%. This variation highlights the complementary nature of the clinical and synthetic datasets in capturing a wide range of vestibular disorders.

In the 50-case test set used to assess peripheral versus central classification, 54% were peripheral, while 46% were central. Among the peripheral cases, the most common diagnoses were BPPV (n = 7), vestibular neuritis (n = 7), and Meniere’s disease (n = 6). Central diagnoses were more heterogeneous and included vertebrobasilar insufficiency (n = 7), vestibular migraine (n = 2), and acoustic neuroma (n = 2).

### Model Performance

**Figure 2.**
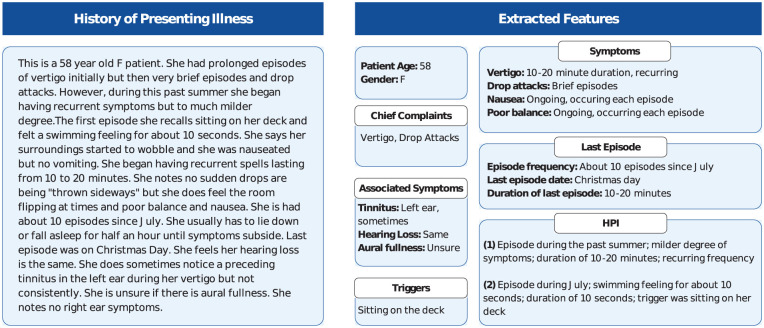
Extracted details from a sample history of presenting illness.

The LLaMA-3.1-8B model successfully extracted key clinical details from broad histories, identifying factors such as age, gender, symptom onset, episode duration and frequency, triggers, and associated symptoms like tinnitus, headaches, and hearing loss (Figure 2). This process occurred without the need for explicit prompts, allowing the model to integrate these features into its diagnostic reasoning. Table 2 outlines the diagnostic performance of the LLaMA-3.1-8B model for PVDs. The model’s top 1 accuracy was 68% for the clinical dataset, 52.5% for the synthetic dataset, and 60.7% for the combined dataset. When considering the top 3 predictions, accuracy improved to 77% in the clinical dataset, 75% in the synthetic dataset, and 71.4% in the combined dataset.

**Table 2.** Performance of the Base LLaMA-3.1-8B Model for Peripheral Vestibular Disease Diagnosis.

Metric	Clinical dataset	Synthetic dataset	Combined dataset
Total cases (N)	100	40	140
Cohen’s kappa for symptom side, κ (SE)	0.74 (0.06)	0.92 (0.05)	0.96 (0.03)
Top 1 symptom side accuracy (%)	86	95	95.7
Cohen’s kappa for diagnosis, κ (SE)	0.10 (0.13)	0.42 (0.10)	0.41 (0.06)
Top 3 diagnostic accuracy (%)	77	75	71.4
Top 1 diagnostic accuracy (%)	68	52.5	60.7
Top 1 accuracy by diagnosis (%)			
BPPV	0	100	90
Labyrinthitis	0	N/A	0
MdDS	N/A	42.9	42.9
Meniere’s disease	85.9	N/A	80.8
SSCD	0	0	0
Vestibular migraines	0	75	76.9
Vestibular paroxysmia	N/A	0	0
Other	0	0	0

Abbreviation: BPPV, benign paroxysmal positional vertigo; MdDS, Mal de Debarquement Syndrome; N/A, not applicable; SSCD, superior semicircular canal dehiscence.

Diagnostic accuracy varied across conditions. BPPV was associated with the highest accuracy of diagnosis (90%), followed by Meniere’s disease (80.8%) and vestibular migraines (76.9%) in the combined dataset. Less common diagnoses, such as superior semicircular canal dehiscence (SSCD) and vestibular paroxysmia, had lower accuracies of 2.9% and 6.4%, respectively. Cohen’s kappa for diagnostic agreement was low in the clinical dataset (κ = 0.10, SE = 0.13), but higher in the synthetic (κ = 0.42, SE = 0.10) and combined datasets (κ = 0.41, SE = 0.06).

Top 1 symptom side accuracy was 86% for the clinical dataset, 95% for the synthetic dataset, and 95.7% for the combined dataset. Cohen’s kappa indicated substantial agreement for the clinical dataset (κ = 0.74, SE = 0.06) and almost perfect agreement for the synthetic (κ = 0.92, SE = 0.05) and combined datasets (κ = 0.96, SE = 0.03). When classifying cases as peripheral versus central etiologies, the model achieved an overall accuracy of 86%. Among the 7 misclassified cases, all involved central etiologies that were incorrectly classified as peripheral. These misclassified cases included vestibular migraine (n = 2), basilar migraine (n = 1), vertebral artery dissection (n = 1), vertebrobasilar insufficiency (n = 1), and multiple sclerosis (n = 1). Cohen’s kappa for peripheral versus central classification indicated substantial agreement (κ = 0.72, SE = 0.10).

## Discussion

The economic burden associated with the diagnosis of dizziness and vertigo is considerable. In 2011, dizziness accounted for approximately 3.9 million emergency department visits, with total costs estimated at $3.9 billion USD.^44^ These figures highlight the growing strain on healthcare systems, particularly in high-demand emergency departments, where clinicians are often required to differentiate between complex PVDs without access to specialized training. Median wait times to see an otolaryngologist extend to 35.2 weeks in Canada.^45^ The integration of LLMs into clinical workflows offers the potential to alleviate these diagnostic bottlenecks. By generating preliminary differential diagnoses, LLMs may facilitate more timely referrals to appropriate specialists, thereby reducing the latency in diagnosis and treatment.

This study is the first to evaluate the diagnostic capabilities of a small-scale, open-source LLM in the field of otolaryngology and is one of the few to explore synthetic data augmentation in clinical datasets. The LLaMa-3.1-8B model demonstrated a top 1 diagnostic accuracy of 60.7% and a top 3 accuracy of 71.4%, with moderate agreement between the LLM and the neuro-otologist. These results compare favorably to the diagnostic accuracies reported in emergency departments, where discrepancies between emergency physicians and otolaryngologists are estimated at 57%. Notably, nearly one-fifth of these discrepancies arise from subclassifications within the dizziness or vertigo category,^46^ underscoring the diagnostic complexity of peripheral vestibulopathies.

Interestingly, the model’s accuracy was higher when tested on purely clinical data compared to synthetic or combined datasets. This discrepancy may be attributed to the increased heterogeneity and representation of rare and atypical presentations in the synthetic dataset, such as superior canal dehiscence syndrome (SCDS). The gap between top 1 and top 3 accuracies was present across all 3 datasets but was more pronounced in the synthetic dataset. While LLaMa-3.1-8B was able to effectively generate a short list of probable diagnoses, it struggled to provide a definitive diagnosis. This limitation may be associated with the synthetic case stems, which were noted to lack detail. In contrast, clinical datasets likely contained more comprehensive patient histories and physical examination findings, enabling the model to perform with greater accuracy.

While previous studies have explored the applications of LLMs in triage, the majority have focused on closed-source models like GPT and Bard.^47,48^ Our LLM demonstrated notable proficiency in extracting key clinical details, including age, gender, symptom onset, episode duration, frequency, triggers, and associated symptoms, from patient histories. The accuracy in these extractions, particularly regarding symptom laterality—where agreement between the LLM and the neuro-otologist was nearly perfect at 95.7%—illustrates the model’s ability to replicate key aspects of clinical reasoning.

### Impact of Synthetic Data Augmentation and Chain-Of-Thought Prompting

Synthetic data augmentation represents a novel approach, particularly for rare disorders such as vestibular migraines and SCDS, which are underrepresented in real-world clinical datasets. More common PVDs—such as vestibular neuritis (10%), BPPV (28%), and Meniere’s disease (13%)—are better represented in medical records, which likely explains the model’s stronger performance in diagnosing these conditions.^49^ However, the model’s lower accuracy in predicting rarer PVDs highlights the challenge of training on limited datasets. Synthetic data augmentation offers a potential solution to help overcome this limitation by providing additional, diverse training examples. Fine-tuning the LLM with synthetic cases may improve its diagnostic precision for less common conditions.

Another key technique employed in this study was chain-of-thought prompting, which breaks down the diagnostic reasoning process into sequential steps, providing the model with additional context at each stage.^40^ This method guides the model to approach complex diagnostic tasks more like a human clinician, where reasoning is often a layered process that involves gathering, analyzing, and synthesizing different pieces of information incrementally. While LLMs are often perceived as black box systems—producing outputs without clear insight into how decisions are made—chain-of-thought prompting enhances both the transparency and interpretability of their decision-making processes. This method allows researchers and clinicians to track the model’s logical progression, making it easier to pinpoint where reasoning errors occur, if present, and to correct them.

### Limitations and Ethical Considerations

The open-source nature of LLMs like LLaMa-3.1-8B offers several advantages, including enhanced transparency and opportunities for model customization in specific clinical contexts. Unlike larger, closed-source models, which may have external dependencies or require third-party cloud services, open-source LLMs allow healthcare institutions to maintain full control over patient data, ensuring compliance with privacy regulations like Health Insurance Portability and Accountability Act or Personal Health Information Protection Act. This control also makes small-scale models more suitable for sensitive clinical applications where data breaches or unauthorized access could pose significant ethical and legal risks.

However, several limitations related to data representation exist, both at the level of our clinical dataset and within the context of model training. Although BPPV is typically the most common peripheral vestibular disorder in the general population, a higher proportion of Meniere’s disease and labyrinthitis was observed in our clinical dataset. This pattern likely reflects local clinical practice patterns and the diagnostic coding conventions employed at the chart extraction site. Specifically, patients diagnosed with Meniere’s disease or labyrinthitis were assigned distinct diagnostic codes, whereas patients presenting with isolated episodic vertigo were often categorized under broader symptom-based codes such as “Not Yet Diagnosed.” This likely contributed to the overrepresentation of certain diagnoses within the dataset and may have influenced model outputs.

Broader concerns regarding representational bias extend to the training of LLMs. These models are often trained on large, heterogeneous datasets that may inadvertently perpetuate existing biases present in medical literature and clinical documentation. These biases can result in unequal diagnostic performance across different demographic groups. For instance, underrepresented populations or conditions may not be as well-represented in the training data, leading to lower accuracy when diagnosing patients from these groups. While synthetic data augmentation can help address some of these gaps, caution is warranted; Without careful prompt engineering and dataset design, synthetic data may inadvertently amplify biases present in the original training data. For example, vestibular migraine accounted for approximately 30% of the synthetic dataset, a proportion likely higher than its true prevalence.^50^ Although larger parameter models may mitigate some of these effects, the lack of public transparency around base model training datasets complicates efforts to systematically correct for such biases. Addressing these concerns in LLMs will require the incorporation of more diverse training datasets, careful fine-tuning, and ongoing efforts to identify and correct for disparities in model outputs.

In addition to these training-related concerns, limitations also exist in the model’s diagnostic reasoning framework. Although we aimed to align outputs with the Bárány Society classification of vestibular disorders, the model was not explicitly prompted to rule out alternative diagnoses, as recommended by the final diagnostic criterion. Instead, it generated a ranked list of 3 differential diagnoses based on likelihood, even when the clinical presentation did not clearly align with a recognized disorder. The workflow also lacked a mechanism for expressing diagnostic uncertainty or acknowledging when no fitting diagnosis could be made.

This becomes particularly important given the stochastic nature of LLM outputs. These models can produce different outputs when presented with identical inputs, introducing variability that is problematic in clinical contexts where consistency and reliability are prioritized. A patient presenting with the same set of symptoms might receive different diagnostic recommendations depending on when the model is queried, raising concerns about the reliability of LLMs as standalone diagnostic tools. We recognize these limitations as key areas for future refinement, particularly to better reflect diagnostic uncertainty and support cautious decision-making. Further work is needed to improve model reproducibility and reduce bias across diverse populations.

### Future Directions

The primary objective of this study was to evaluate the model’s ability to support diagnostic reasoning based on history-taking alone, particularly in primary care settings where access to specialized vertigo assessments or diagnostic equipment is often limited. The intent was not to replace otolaryngologists, but rather to assist in early symptom triage and guide appropriate referrals. In our current design, the model generates a ranked list of differential diagnoses with accompanying justifications and allows users to adjust the number of differentials produced through prompting. While this study focused primarily on peripheral vertigo causes, it is likely that expanding the diagnostic scope to include a broader range of vestibular and non-vestibular etiologies will be clinically important to maximize the tool’s relevance across different care environments.

As LLMs become more integrated into clinical workflows, ongoing validation and monitoring of their performance will be essential. Clinicians will need to be trained to interpret and critically appraise model outputs, ensuring that human oversight remains a crucial component of the diagnostic process. In parallel, evaluating the cost-effectiveness of LLMs, not only in terms of financial impact, but also in improving patient outcomes and diagnostic efficiency, will be important for informing broader adaptation.

Future technical improvements, including fine-tuning the model on synthetic and clinical datasets, and optimizing prompt design will be critical to maximizing model performance and usability. Expanding LLM applications to support real-time history-taking, and integration of physical examination findings, laboratory investigations, and imaging results into model inputs, may further enhance diagnostic accuracy. Ultimately, continued iteration and clinical evaluation will be key to realizing the full potential of LLMs in supporting diagnostic reasoning in both generalist and specialist care settings.

## Conclusion

This study demonstrates the potential of small-scale LLMs, such as LLaMA-3.1-8B, in diagnosing otolaryngologic conditions such as peripheral vestibulopathies. Our findings suggest that synthetic data augmentation and advanced reasoning techniques, including prompt chaining, improve the model’s generalizability and accuracy. Nonetheless, challenges remain in optimizing diagnostic precision. Future research should focus on fine-tuning models with larger, more heterogeneous datasets and exploring their application in clinical settings, such as with real-time patient history-taking. With continued refinement, LLMs can enhance diagnostic workflows, reduce specialist wait times, and lower healthcare costs.
